# Idarubicin‐Loaded DEB‐TACE plus Lenvatinib versus Lenvatinib for patients with advanced hepatocellular carcinoma: A propensity score‐matching analysis

**DOI:** 10.1002/cam4.4937

**Published:** 2022-06-13

**Authors:** Wenzhe Fan, Bowen Zhu, Shufan Yue, Xinlin Zheng, Xinhua Zou, Fuliang Li, Liangliang Qiao, Yanqin Wu, Miao Xue, Hongyu Wang, Yiyang Tang, Jiaping Li

**Affiliations:** ^1^ Department of Interventional Oncology Sun Yat‐sen University First Affiliated Hospital Guangzhou China; ^2^ Department of Ultrasonic Sun Yat‐sen University First Affiliated Hospital Guangzhou China; ^3^ Liver and Gall Surgical Department Gaozhou People's Hospital Gaozhou China; ^4^ Department of Oncology Jinshazhou Hospital of Guangzhou University of Chinese Medicine Guangzhou China

**Keywords:** carcinomas, chemoembolization, hepatocellular, idarubicin, lenvatinib, therapeutic

## Abstract

**Aims:**

To investigate the efficacy and safety of lenvatinib and idarubicin‐loaded drug‐eluting beads transarterial chemoembolization (IDADEB‐TACE) in primary advanced hepatocellular carcinoma (HCC).

**Methods:**

This retrospective study included patients with primary advanced HCC who received either lenvatinib monotherapy or lenvatinib plus IDADEB‐TACE as first‐line treatment from September 2019 to September 2020 at three institutes. Overall survival (OS), time to progression (TTP), objective response rate (ORR), and adverse events were compared. Propensity score‐matching was used to reduce the influence of confounding factors on the outcomes.

**Results:**

The study reviewed 118 patients who received lenvatinib plus IDADEB‐TACE (LIDA group) and 182 who received lenvatinib alone (LEN group). After propensity score‐matching, 78 pairs of patients remained. Compared to patients in the LEN group, those in the LIDA group had better post‐treatment ORR (57.7% vs. 25.6%, *p* < 0.001, respectively), median OS and TTP (15.7 vs. 11.3 months, hazard ratio [HR] = 0.50, *p* < 0.001; 8.0 vs. 5.0 months, HR = 0.60, *p* = 0.003, respectively), 6‐ and 12‐month OS rates (88.5% vs. 71.4%; 67.6% vs. 43.4%, respectively), and progression‐free rates at 6 and 12 months (60.3% vs. 42.3%; 21.1% vs. 10.3%, respectively). Vascular invasion, α‐fetoprotein level, and treatment type were independent OS predictors, and vascular invasion and treatment type were independent TTP predictors. Incidences of nausea/vomiting, fever, abdominal pain, and increased ALT/AST were higher in the LIDA group than in the LEN group.

**Conclusions:**

Lenvatinib plus IDADEB‐TACE is well‐tolerated and more effective than lenvatinib monotherapy in patients with advanced HCC.

## Graphical abstract

Idarubicin‐loaded DEB‐TACE plus lenvatinib showed improved overall survival and time to progression compared with lenvatinib monotherapy.

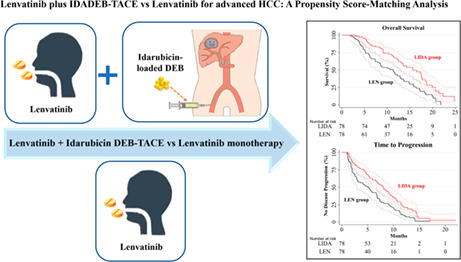

## 1 INTRODUCTION

Hepatocellular carcinoma (HCC) has the fifth high morbidity and second cancer‐related mortality around the world.^1^ In China, many patients with HCC were diagnosed at advanced stage and characterized by vascular invasion or extrahepatic metastasis and poor prognosis.^2^ First‐line systemic therapy with lenvatinib is one of the standard treatments for Barcelona Clinic Liver Cancer (BCLC) stage C HCC.^3^, ^4^ The modest efficacy of this targeted therapy confers limited long‐term survival benefit, as the objective response rate (ORR) was only 24.1% and median overall survival was 13.6 months in REFLECT trial.^5^ The primary cause of mortality in patients with advanced HCC is intrahepatic tumor progression rather than extrahepatic metastatic disease^6^, ^7^; hence, local therapies such as transarterial chemoembolization (TACE) that decrease the intrahepatic tumor burden rapidly, enhances the anti‐tumor effect of tyrosine kinase inhibitor (TKI) targeted drugs in patients with advanced HCC.^8^ Several meta‐analyses of retrospective studies, revealed that sorafenib, a first‐generation TKI drug, combined with TACE had significant survival benefit when compared with sorafenib alone in patients with unresectable HCC^9^, ^10^; however, the prospective STAH trials showed that sorafenib plus TACE had better objective response rate (ORR) or progression‐free survival, but failed to improve overall survival (OS) of patients with advanced HCC when compared with sorafenib monotherapy.^11^ While lenvatinib plus TACE has been reported to possess a manageable safety profile and a possibility of promising efficacy in advanced HCC,^12^, ^13^ no‐superiority for OS to sorafenib plus TACE was observed.^14^ To data, there is no study comparing lenvatinib plus TACE with lenvatinib monotherapy to prove the efficacy of combined treatment. Therefore, the application of this combination still needs further investigations and developments that might possess the potential of long‐term efficacy owing to TACE.

The drugs and embolic agents associated with arterial chemoembolization likely restrict the localized effect of TACE. Drug‐eluting beads (DEB), a uniform embolic material loaded with a water‐soluble drug, selectively delivers specific amounts of chemotherapeutic agents to the target lesion for a relative long period, minimizing the systemic blood concentration and related systemic effects. The result of randomized controlled trials has revealed the similar benefit of progression‐free survival and OS by comparing DEB‐TACE and conventional TACE (cTACE) therapy, where DEB‐TACE is known to increase the ORR significantly and reduce TACE‐related liver damage and severe adverse events (AEs).^15^, ^16^ Lipiodol cTACE might further reduce the liver function reserve already limited in patients with advanced HCC, thereby decreasing the duration of systemic therapy significantly.^17^ Hence, DEB‐TACE has shown superior or at least similar outcomes compared to cTACE, exhibiting a safety profile in patients with severe HCC at an advanced stage.

In the drug series, currently, the most commonly used drug in TACE is doxorubicin, despite the lack of strong evidence except for a single‐arm phase II trial that supported its efficacy in HCC.^18^ Recent research has suggested that idarubicin had the highest cytotoxicity and was significantly more effective than 10 other agents, including doxorubicin, cisplatin, and epirubicin in *in vitro* screening of antitumor drugs for TACE.^19^ The high cytotoxicity of idarubicin could be due to its lipophilic nature, which explains its efficient penetration through the lipid bilayer of tumor cell membranes.^20^, ^21^ The LIDA‐B phase I^22^ and IDASPHERE phase II^23^ trials have shown promising results (the best ORR, 68%; and OS, 18.6 months) for idarubicin treated unresectable HCC when used in chemolipiodolization. Therefore, given these encouraging local effects, idarubicin‐loaded DEB‐TACE (IDADEB‐TACE) might exert better synergistic effects with systemic lenvatinib treatment on advanced HCC than cTACE.

However, literatures directly compared lenvatinib plus DEB‐TACE with lenvatinib monotherapy as first‐line treatment were limited. Therefore, the primary aim of our study was to assess the efficacy and safety of first‐line lenvatinib plus DEB‐TACE loaded idarubicin versus that of lenvatinib alone in patients with advanced HCC.

## 2 METHODS

### 2.1 Study design and case enrollment

This was a multicenter, retrospective cohort study including patients with advanced HCC treated at three institutes from September 2019 to September 2020. The study was approved by the Institutional Review Board of Sun Yat‐sen University First Affiliated Hospital (Ethical number: [2020]256) and informed consent was waived because this is a retrospective study.

The eligibility criteria were: (a) Age 18–75 years; (b) advanced primary HCC (consistent with the EASL/AASLD criteria^3^, ^4^) without receipt of any previous treatment; (c) Child‐Pugh score A5‐B9; (d) BCLC stage C; (e) good hematologic function (platelet count no less than 60 × 10^9^/L, hemoglobin concentration no <85 g/L, prothrombin time no more than 6 s above the upper limit of normal); (f) Eastern Cooperative Oncology Group (ECOG) performance status score of 0–1; (g) acceptable renal function: Serum creatinine < 1.5 × upper limit of normal.

The exclusion criteria were: Hepatic decompensation, such as hepatic encephalopathy and esophageal or gastric variceal bleeding; central nervous system metastases; portal vein tumor thrombus (PVTT) in the main portal vein; other malignant diseases; contraindications for TACE, such as portal‐systemic shunt, hepatofugal flow, or obvious atherosclerosis.

### 2.2 Treatment

Oral lenvatinib (Lenvima; Eisai Co., Ltd., Tokyo, Japan) treatment (8 mg/day [<60 kg] or 12 mg/day [≥60 kg]) was started, and the dose was reduced (to 8 mg/day, 4 mg/day, or 4 mg every other day) because of lenvatinib‐related toxicities until the AEs were alleviated or eliminated. If the AEs continued even after dose adjustment, lenvatinib treatment was interrupted until it alleviated or disappeared.

The first DEB‐TACE procedure was performed between 7 and 14 days after the first administration of lenvatinib. After imaging and catheterization of hepatic arteries with standard angiographic protocols and equipment to visualize HCC blood supply, selective catheterization was performed to achieve lobar or segmental chemoembolization. Lenvatinib was continuously administered without interruption during DEB‐TACE.

DEB‐TACE was performed as previously recommended.^24^ We used DC Beads (Biocompatibles UK) or CalliSpheres microspheres (Jiangsu Hengrui Medicine Co., Ltd., Jiangsu, China) with diameters of 100–300 or 300–500 μm. Each vial of these particles (2 ml) was added to 10 mg of idarubicin (Pfizer) with sterilized water for 30 min. The DEB dose was determined by tumor volume (calculation of ellipsoid volume: height × width × length × π/6). The endpoint of primary chemoembolization was complete devascularization of the tumor confirmed via angiography.^24^ Figure S1 shows a typical patient.

### 2.3 Data collection

Patients were followed up at 4–6 weeks. Serum levels of α‐fetoprotein (AFP) and liver enzymes were measured. Triphasic contrast‐enhanced CT of upper abdomen was performed at baseline before commencing treatment and every 4–6 weeks during the first 6 months after every treatment session. Target lesion responses were assessed by two independent radiologists blinded to each other's evaluation; any inconsistencies were resolved by discussion.

### 2.4 Outcomes and adverse events

Tumor response, OS, time to progression (TTP), and AEs were assessed. Local tumor response was assessed according to images acquired 4 weeks after treatment using the modified Response Evaluation Criteria in Solid Tumors.^25^ Local response was graded as complete response (CR), partial response (PR), stable disease (SD), and progression disease (PD). And objective response rate (ORR) was defined as the sum of CR and PR, and disease control rate (DCR) as the sum of CR, PR, and SD. Measurements were performed by two blinded, independent radiologists from the Department of Medical Imaging. A third blinded, experienced radiologist reviewed the results when there was uncertainty or disagreement. If a new lesion or recurrence was detected during follow‐up, the doctor who treated this patient for the first time will review the patient's medical history and examination results, and design the re‐treatment program under full consideration of patients' liver function and tumor burden. DEB‐TACE was repeated at variable time intervals to avoid progressive liver dysfunction and hepatic artery damage.^26^ Therapeutic indications and patient tolerance were assessed according to the inclusion criteria before each new course, and repeated courses of TACE were planned on‐demand. OS was defined as the time from the day of initial lenvatinib administration and death due to any cause. TTP was the interval between the day of initial lenvatinib administration until the detection of tumor progression. Patients lost to follow‐up were censored on the date of their last follow‐up. AEs were scaled according to the Common Terminology Criteria of Adverse Events v4.0.^27^ The last follow‐up date was December 31, 2021.

### 2.5 Statistical analysis

Programming and statistical analyses were performed using STATA version 15.0 software (Stata Corporation, College Station, TX, USA) and R version 4.0.2 (Stanford University, CA, USA). Continuous variables were expressed as the mean ± standard deviation, and between‐group comparisons were performed using the Student's *t*‐test. Categorical data were presented as frequencies, and between‐group differences were evaluated using Pearson's chi‐squared test. The propensity score model included variables such as age, gender, alpha‐fetoprotein (AFP) level, hepatitis B virus (HBV) infection, ECOG score, Child‐Pugh class, main tumor size, vascular invasion (VI), and extrahepatic spread (EHS). A 1:1 matched analysis using nearest‐neighbor matching with a caliper width of 0.3 without replacement was performed to deduce matched pairs from two groups based on the estimated propensity score. Survival data, including OS and TTP, were estimated by the Kaplan–Meier method and the comparison was performed using log‐rank test. Univariate and multivariate analyses were conducted using the log‐rank test and Cox regression analysis for variables, with clinical significance and *p* < 0.1 in univariate analyses. All statistical tests were two‐sided, and *p* < 0.05 were considered significant.

## 3 RESULTS

### 3.1 Baseline characteristics

**FIGURE 1:**
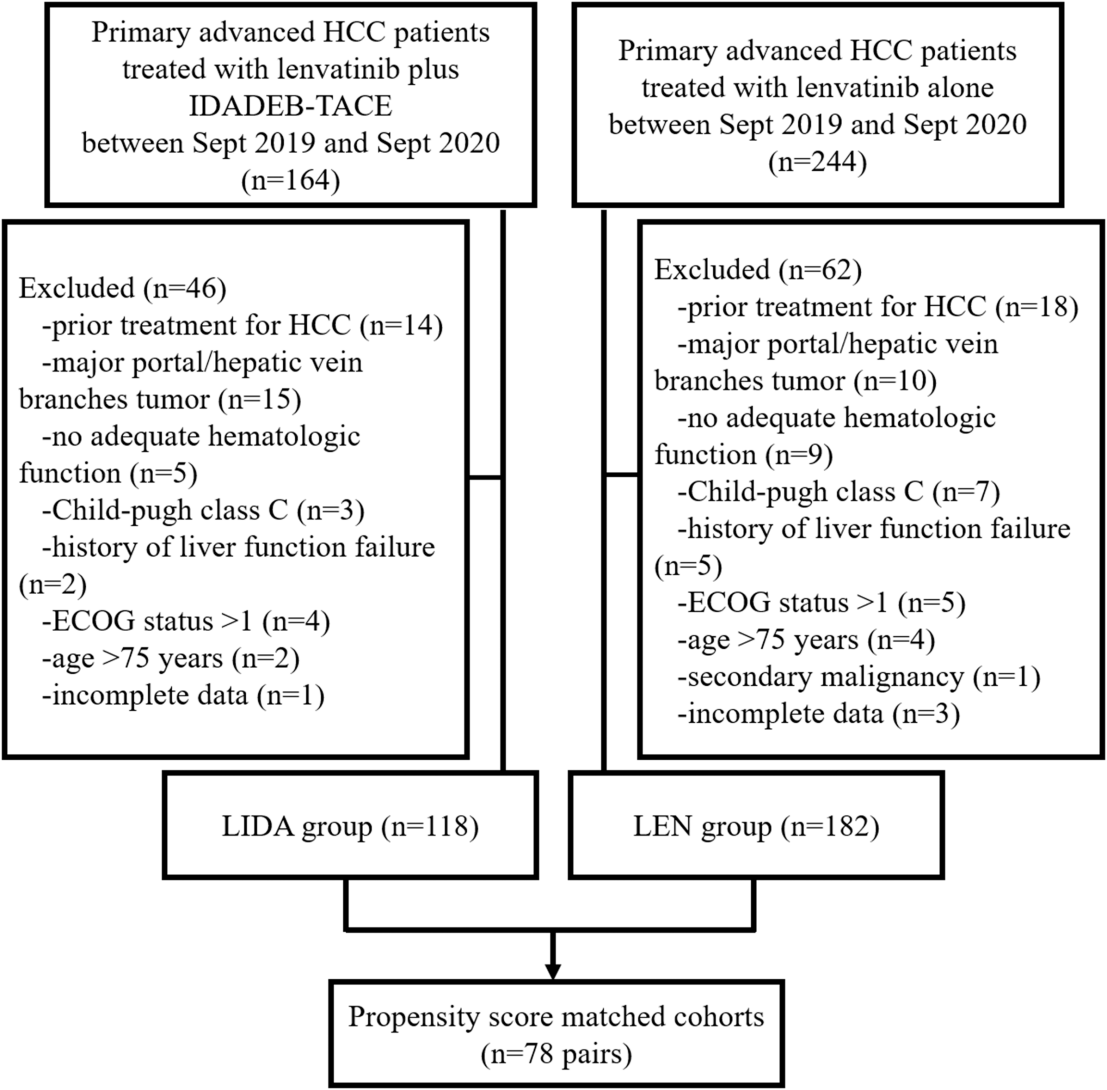
Flowchart showing the selection of patients

**FIGURE 2:**
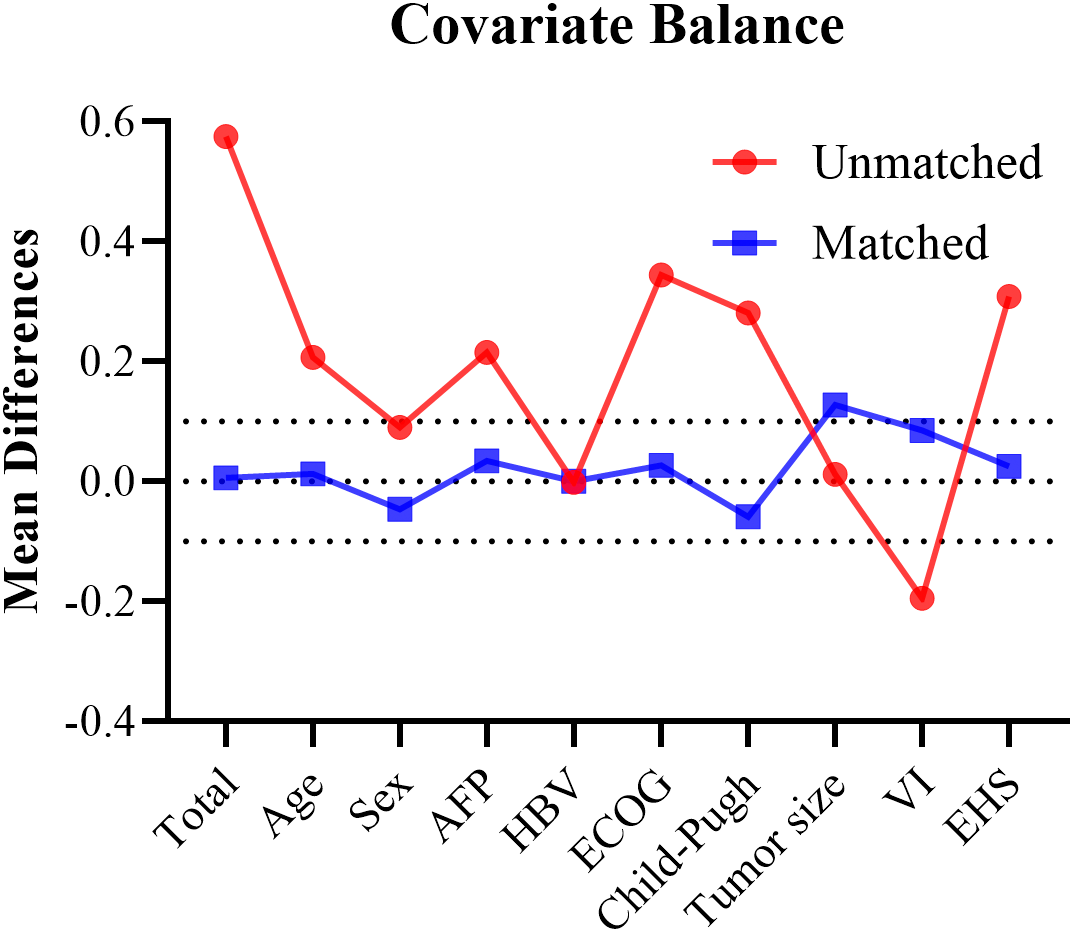
Mean differences in covariates between patients with advanced hepatocellular carcinoma receiving lenvatinib plus idarubicin‐loaded DEB‐TACE or lenvatinib alone before and after propensity score‐matching analysis. Abbreviations: AFP, alpha‐fetoprotein; ECOG, Eastern Cooperative Oncology Group (performance status); EHS, extrahepatic spread; HBV, hepatitis B virus; VI, vascular invasion.

Overall, 300 patients were enrolled, out of which 118 were treated with lenvatinib plus idarubicin‐loaded DEB‐TACE (LIDA group) and 182 with lenvatinib alone (LEN group) (Figure 1). The baseline characteristics of two groups are summarized in Table 1. Before propensity score‐matching (PSM), patients in the LIDA group had a higher ECOG score (*p* = 0.001), AFP level (*p* = 0.009), and Child‐Pugh class (*p* = 0.007), less were found with VI (*p* = 0.078) and more with EHS (*p* = 0.008), compared with those in the LEN group. After PSM, matched cohorts of 78 patients per group were extracted, with well‐balanced baseline characteristics (shown in Table 1). Covariate balance between the two groups before and after the PSM was assessed using the love, jitter, histogram, and QQ plots. (Figure 2; Figures S2–S4).

**TABLE 1:** Baseline characteristics

Characteristics	Before PSM			After PSM		
	LEN group (*n* = 182)	LIDA group (*n* = 118)	*p* value	LEN group (*n* = 78)	LIDA group (*n* = 78)	*p* value
Age (y)	49 ± 11	52 ± 11	0.072	51 ± 11	51 ± 10	0.933
<50 (*n*, %)	86 (47.3)	47 (39.8)	0.206	31 (39.7)	33 (42.3)	0.745
≥50 (*n*, %)	96 (52.7)	71 (60.2)		47 (60.3)	45 (57.7)	
Gender (*n*, %)			0.478			0.786
Male	162 (89.0)	108 (91.5)		71 (91.0)	70 (89.7)	
Female	20 (11.0)	10 (8.5)		7 (9.0)	8 (10.3)	
Body weight (kg)			0.432			0.618
<60 (*n*, %)	73 (40.1)	42 (35.6)		27 (34.6)	30 (38.5)	
≥60 (*n*, %)	109 (59.9)	76 (64.4)		51 (65.4)	48 (61.5)	
HBV (*n*, %)			0.994			1.000
Absence	20 (11.0)	13 (11.0)		11 (14.1)	11 (14.1)	
Presence	162 (89.0)	105 (89.0)		67 (85.9)	67 (85.9)	
AFP (ng/ml)	17,109 ± 86,562	69,225 ± 242,639	0.009	6759 ± 19,838	15,048 ± 42,787	0.122
<400	96 (52.7)	64 (54.2)	0.800	48 (61.5)	46 (59.0)	0.744
≥400	86 (47.3)	54 (45.8)		30 (38.5)	32 (41.0)	
ECOG score (*n*, %)			0.001			0.853
0	150 (82.4)	78 (66.1)		59 (75.6)	58 (74.4)	
1	32 (17.6)	40 (33.9)		19 (24.4)	20 (25.6)	
Child‐Pugh class			0.007			0.667
A	158 (86.8)	88 (74.6)		64 (82.1)	66 (84.6)	
B	24 (13.2)	30 (25.4)		14 (17.9)	12 (15.4)	
Intrahepatic tumors number (*n*, %)			0.669			0.565
Single	36 (19.8)	21 (17.8)		19 (24.4)	16 (20.5)	
Multiple	146 (80.2)	97 (82.2)		59 (75.6)	62 (79.5)	
Main tumor size (cm, %)	8.0 ± 4.0	8.0 ± 4.8	0.911	7.6 ± 4.0	8.2 ± 5.1	0.395
<5	45 (24.7)	36 (30.5)	0.270	22 (28.2)	24 (30.8)	0.725
≥5	137 (75.3)	82 (69.5)		56 (71.8)	54 (69.2)	
VI (*n*, %)			0.078			0.585
Yes	147 (80.8)	85 (72.0)		56 (71.8)	59 (75.6)	
No	35 (19.2)	33 (28.0)		22 (28.2)	19 (24.4)	
EHS (*n*, %)			0.008			0.873
Yes	66 (36.3)	57 (48.3)		37 (47.4)	38 (48.7)	
No	116 (63.7)	61 (51.7)		41 (52.6)	40 (51.3)	

Abbreviations: AFP, alpha‐fetoprotein; ECOG, Eastern Cooperative Oncology Group (performance status); EHS, extrahepatic spread; HBV, hepatitis B virus; LIDA group, patients treated with lenvatinib plus idarubicin‐loaded drug‐eluting beads transarterial chemoembolization; LEN group, patients treated with lenvatinib alone; PSM, propensity score matching; VI, vascular invasion.

### 3.2 Tumor response, OS, and TTP

During follow‐up, 57.6% (68/118) of the patients in the LIDA group and 84.1% (153/182) in the LEN group died. In the LIDA group, 9 (11.5%), 36 (46.2%), 14 (17.9%), and 19 (24.4%) patients exhibited CR, PR, SD, and PD, respectively (Table 2). The ORR in the LIDA group were higher than those in the LEN group (57.7% vs. 25.7%, *p* < 0.001), as well as disease control rate (75.6% vs. 56.4%, *p* = 0.011).

**TABLE 2:** Best response after treatment

Variable	Group, No (%)		*p* value
	LIDA group (*n* = 78)	LEN group (*n* = 78)	
Complete response	9 (11.5%)	2 (2.6%)	0.029
Partial response	36 (46.2%)	18 (23.1%)	0.002
Stable disease	14 (17.9%)	24 (30.8%)	0.062
Progressive disease	19 (24.4%)	34 (43.6%)	0.011
Objective response rate	45 (57.7%)	20 (25.6%)	<0.001
Disease control rate	59 (75.6%)	44 (56.4%)	0.011

*Note*: Objective response rate = complete response rate + partial response rate; Disease control rate = complete response rate + partial response rate + stable disease rate.

**FIGURE 3:**
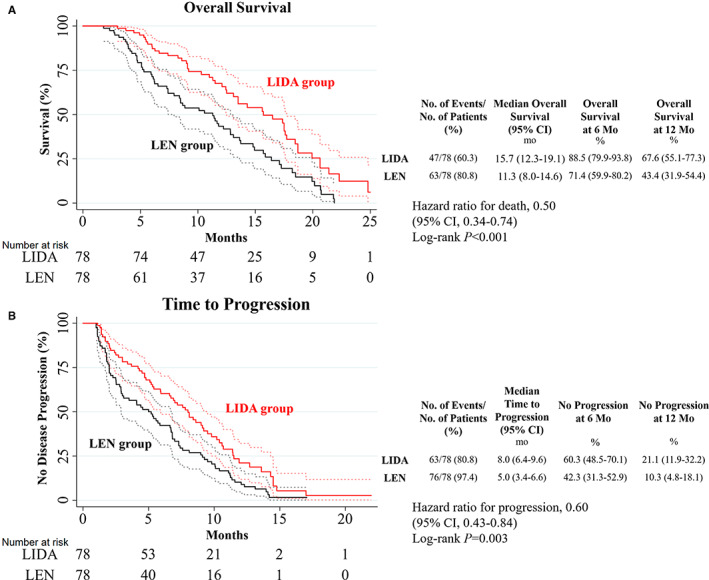
(A) Overall survival and (B) time to progression in patients in the LIDA and LEN groups. Dotted lines represent the 95% confidence interval. Abbreviations: CI, confident interval; LIDA group, patients treated with lenvatinib plus idarubicin‐loaded drug‐eluting beads transarterial chemoembolization; LEN group, patients treated with lenvatinib alone.

The median OS in the LIDA group (15.7 months, 95% confidence interval [CI]: 12.3–19.1) was longer than in the LEN group (11.3 months, 95% CI: 8.0–14.6) (hazard ratio [HR] = 0.50, 95% CI: 0.34–0.74, *p* < 0.001). The survival rates at 6 and 12 months were 88.5% (95% CI: 79.9–93.8) and 67.6% (95% CI: 55.1–77.3) in the LIDA group and 71.4% (95% CI: 59.9–80.2) and 43.4% (95% CI: 31.9–54.4) in the LEN group, respectively (Figure 3A). The median TTP in the LIDA group (8.0 months, 95% CI: 6.4–9.6) was longer than in the LEN group (5.0 months, 95% CI: 3.4–6.6) (HR = 0.60, 95% CI: 0.43–0.84, *p =* 0.003). The no‐progression‐rates at 6 and 12 months were 60.3% (95% CI: 48.5–70.1) and 21.1% (95% CI: 11.9–32.2) in the LIDA group and 42.3% (95% CI, 31.3–52.9) and 10.3% (95% CI: 4.8–18.1) in the LEN group, respectively (Figure 3B).

### 3.3 Subgroup analysis

**FIGURE 4:**
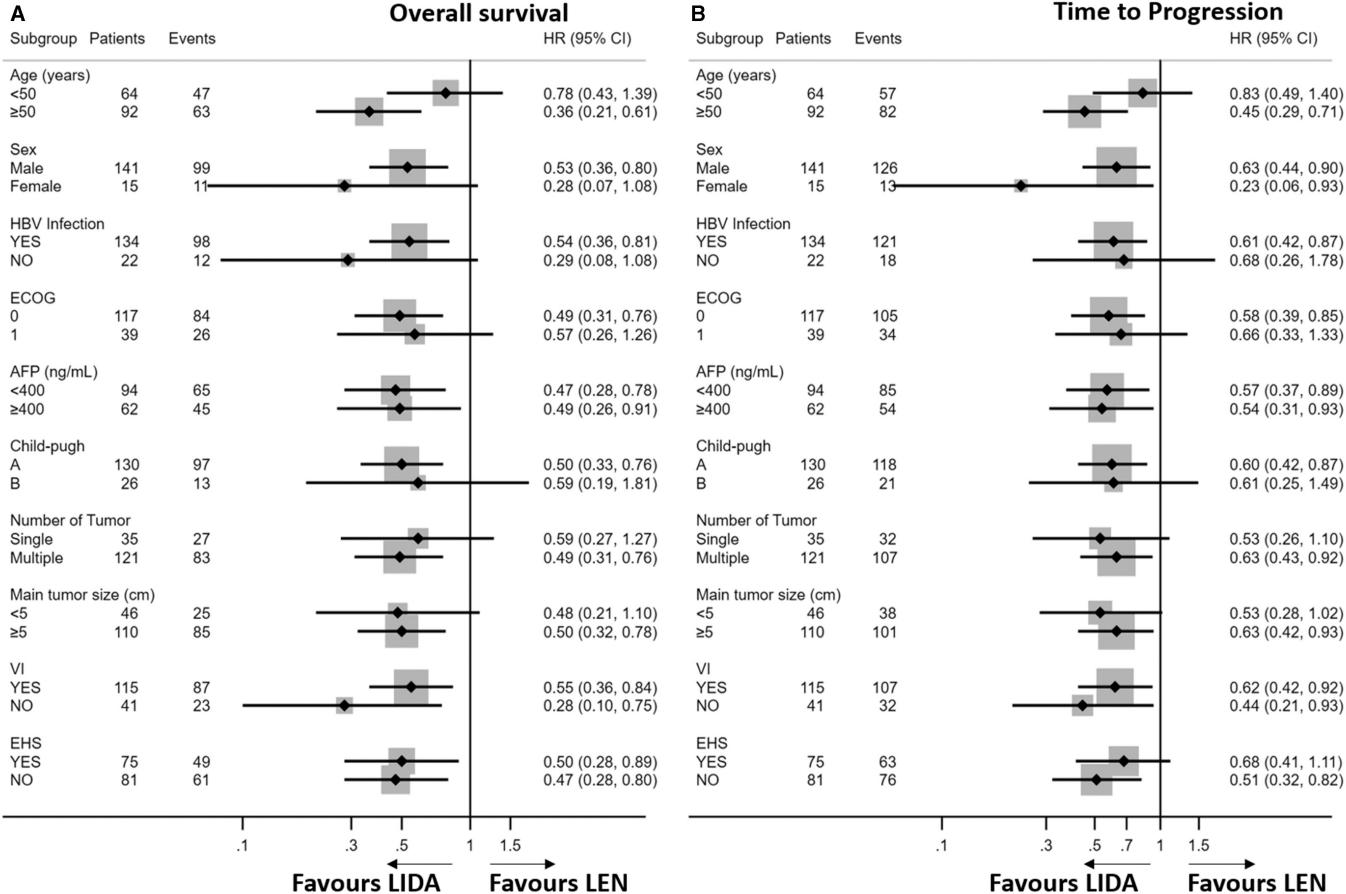
Subgroup analyses of (A) overall survival and (B) time to progression in the patient subgroups. Abbreviations: AFP, α‐fetoprotein; CI, confidence interval; ECOG‐PS, Eastern Cooperative Oncology Group (performance status); HBV, hepatitis B virus; HR, hazard ratio; VI vascular invasion.

The median OS of the following patient categories was longer in the LIDA group than in the LEN group: Age ≥ 50 years, male, with HBV infection, ECOG score 0, AFP level < or ≥400 ng/mL, Child‐Pugh class A, multiple intrahepatic tumors, main tumor size >5 cm, with or without VI status, and with or without EHS status (Figure 4A). TTP in the LIDA group was significantly longer than in the LEN group for patients over 50 years old, men or women, HBV‐infected, ECOG score 0, AFP level < or ≥400 ng/ml, multiple intrahepatic tumors, with or without VI status, and without EHS status (Figure 4B).

The median OS and TTP of patients with first‐order PVTT were similar in the two groups (*p* = 0.318 and *p* = 0.152, respectively; Figure S5A,B); however, the median OS and TTP of patients with second‐ or lower‐order PVTT was significantly higher in the LIDA group than in the LEN group (HR = 0.40, *p* = 0.005; HR = 0.56, *p* = 0.039, respectively) (Figure S5C,D).

### 3.4 Prognostic factors for OS and TTP

Univariate analysis showed that AFP level (*p* = 0.011), VI (*p* = 0.039), and treatment type (*p<* 0.001) were significantly correlated with OS, whereas AFP level (*p* = 0.060), VI (*p* = 0.003), and treatment type (*p =* 0.003) were significant prognostic factors for TTP. In multivariate cox regression analysis, AFP level, VI, and treatment type were independent predictors of OS (HR = 1.68, *p* = 0.010; HR = 1.66, *p =* 0.034; HR = 0.46, *p* < 0.001, respectively), and only VI and treatment type were independent predictors of TTP (HR = 1.87, *p =* 0.003; HR = 0.57, *p* = 0.001, respectively) (Table 3).

**TABLE 3:** Uni‐ and multivariate cox regression analyses of prognostic factors associated with overall survival and time to progression

Factor	Overall survival					Time to progression				
	Univariate		Multivariate			Univariate		Multivariate		
	HR	*p*‐value	HR	95% CI	*p*‐value	HR	*p*‐value	HR	95% CI	*p*‐value
Gender	0.62	0.141				0.65	0.141			
Age^†^	1.01	0.954				1.14	0.456			
HBV	1.41	0.259				1.27	0.352			
ECOG PS score	1.25	0.330				1.26	0.247			
AFP^‡^	1.65	0.011	1.68	1.13–2.50	0.010	1.39	0.060	1.39	0.98–1.96	0.067
Child Pugh class	1.13	0.678				1.27	0.324			
Intrahepatic tumor number^§^	0.94	0.784				0.98	0.925			
Main tumor size^¶^	1.31	0.243				1.08	0.705			
VI	1.63	0.039	1.66	1.04–2.65	0.034	1.84	0.003	1.87	1.24–2.80	0.003
EHS	1.04	0.850				0.87	0.420			
Treatment^#^	0.50	<0.001	0.46	0.31–0.67	<0.001	0.60	0.003	0.57	0.41–0.80	0.001

*Note*: The cut‐off value is ^†^ 50 years old; ^‡^ 400 ng/mL; ^§^ ≥ 2; ^¶^ 5 cm; ^#^ LEN group as reference.

Abbreviations: AFP, alpha fetoprotein; CI, confidence interval; ECOG, Eastern Cooperative Oncology Group (performance status); EHS, extrahepatic spread; HBV, hepatitis B virus; HR, hazard ratio; VI, vascular invasion.

### 3.5 AEs

The median duration of lenvatinib treatment was 7.5 months in the LIDA group and 5.8 months in the LEN group. The following AEs of all grades were more frequent in the LIDA group: Nausea/vomiting (24 [30.8%] vs. 11 [14.1%], *p* = 0.013), fever (27 [34.6%] vs. 4 [5.1%], *p <* 0.001), abdominal pain (40 [51.3%] vs. 17 [21.8%], *p <* 0.001), and increased ALT/AST (19 [24.4%] vs. 8 [10.3%], *p* = 0.020) (Table 4). The only AE of grade ≥ 3 which occurred more commonly in the LIDA group was increased ALT/AST (9 [11.5%] vs. 1 [1.3%], *p* = 0.009). No unexpected AEs or drug‐related deaths were observed. Eighteen (23.1%) patients in the LIDA group and 15 (19.2%) in the LEN group (*p* = 0.556) received a dose reduction because of the severe AE. The interruption of lenvatinib administration occurred in 14 (17.9%) patients in the LIDA group, and 13 (16.7%) in the LEN group (*p* = 0.832). The most common cause of dose reduction or interruption was hypertension (Table S1).

**TABLE 4:** Adverse events in the LIDA and LEN groups

Adverse event	All grades			Grade ≥3		
	Group, No (%)		*p* value	Group, No (%)		*p* value
	LIDA group (*n* = 78)	LEN group (*n* = 78)		LIDA group (*n* = 78)	LEN group (*n* = 78)	
Hand‐foot skin reaction	24 (30.8%)	22 (28.2%)	0.725	3 (3.8%)	3 (3.8%)	1.000
Diarrhea	36 (46.2%)	34 (43.6%)	0.747	4 (5.1%)	3 (3.8%)	0.699
Fever	27 (34.6%)	4 (5.1%)	<0.001	1 (1.3%)	0	0.316
Decreased appetite	32 (41.0%)	32 (41.0%)	1.000	4 (5.1%)	3 (3.8%)	0.699
Hypertension	51 (65.4%)	49 (62.8%)	0.739	17 (21.8%)	15 (19.2%)	0.692
Abdominal pain	40 (51.3%)	17 (21.8%)	<0.001	3 (3.8%)	1 (1.3%)	0.311
Nausea/Vomiting	24 (30.8%)	11 (14.1%)	0.013	2 (2.6%)	1 (1.3%)	0.560
Weight decreased	27 (34.6%)	30 (38.5%)	0.618	5 (6.4%)	6 (7.7%)	0.754
Rash	10 (12.8%)	9 (11.5%)	0.807	1 (1.3%)	0	0.316
Fatigue	26 (33.3%)	24 (30.8%)	0.732	3 (3.8%)	2 (2.6%)	0.649
Dysphonia	20 (25.6%)	20 (25.6%)	1.000	0	1 (1.8%)	0.316
Proteinuria	28 (35.9%)	24 (30.8%)	0.497	4 (5.1%)	4 (5.1%)	1.000
ALT/AST increased	19 (24.4%)	8 (10.3%)	0.020	9 (11.5%)	1 (1.3%)	0.009
Hyperbilirubinemia	18 (23.1%)	17 (21.8%)	0.848	5 (6.4%)	5 (6.4%)	1.000
Constipation	14 (17.9%)	13 (16.7%)	0.832	1 (1.3%)	1 (1.3%)	1.000

*Note*: Data are *n* (%). Only adverse events occurred in no less than 10% patients of each group were listed.

Abbreviations: ALT, alanine aminotransferase; AST, aspartate aminotransferase; LEN group, patients treated with lenvatinib alone; LIDA group, patients treated with lenvatinib plus TACE.

## 4 DISCUSSION

The current study initially evaluated the efficacy and safety of lenvatinib plus IDADEB‐TACE treating patients with BCLC stage C HCC and found that the combined therapy improved OS, TTP, and ORR compared with lenvatinib alone. The median OS in the LEN group of our study was comparable to that of the BCLC stage C subgroup in the REFLECT trial (11.8 months).^5^ Moreover, the current survival results of patients treated with lenvatinib plus IDADEB‐TACE are similar to that of a prospective study on unresectable BCLC stage A–C HCC treated by doxorubicin‐loaded DEB‐TACE plus sorafenib (TACE‐2), which reported TTP of 7.9 months,^28^ and better than those trials on advanced HCC with PVTT treated with conventional TACE plus lenvatinib (OS: 14.5 months, TTP: 4.7 months)^14^ or conventional TACE plus sorafenib (OS of 12.8 months, TTP of 5.3 months).^11^ In addition, TTP rate at 6 months and the OS rate at 12 months (60.3% and 67.6%) in our study were similar to those observed with atezolizumab‐bevacizumab combination therapy, reported as the optimal systemic treatment for unresectable HCC in the IMbrave150 trial (54.5% and 67.2%).^29^ The ORR was higher in our study (57.7%) than that reported in IMbrave150 trial (33.3%),^29^ indicating a mutual benefit and enhanced local effect of lenvatinib plus IDADEB‐TACE. Although the analysis of data in IMbrave150 trial for advanced HCC was not stratified, the combination of TACE‐local and TKI‐systemic treatment in our study achieved a similar effect to that of targeted drugs combined with immunotherapy, indicating that IDADEB‐TACE plus lenvatinib can be used as an effective choice for the treatment of advanced HCC.

The reasons for the improved efficacy of lenvatinib in combination with IDADEB‐TACE were as follows. First, the intrahepatic tumor burden, a key negative factor for the low survival rate of patients with HCC, is abundantly reduced by IDADEB‐TACE.^30^ Owing to the lipophilic nature of idarubicin, it could efficiently penetrate through the lipid bilayer of tumor cell membranes, altering the stability of a highly concentrated aqueous solution stored in polypropylene syringes, which makes it more conducive to the anti‐tumor effect of TACE.^31^ Several studies have reported high tumor response rates to idarubicin‐loaded chemoembolization with best ORR of 68% and complete response rate of 39% in 78% BCLC stage B HCC patients, which may result in significantly longer TTP than that of doxorubicin,^22^, ^23^ suggesting that IDADEB‐TACE promotes the systemic effect of lenvatinib to effectively control overall tumor burden. Second, idarubicin and TKI exhibited a synergistic effect, as demonstrated in the treatment of acute myeloid leukemia.^32^ Multi‐drug resistant tumor cells exhibit a short‐term response to TKI targeted drugs but are more susceptible to idarubicin because of its unique lipophilic properties and enhanced uptake,^33^ which would help prolong the anti‐tumor effect of lenvatinib. Third, as a localized therapy, IDADEB‐TACE, which preserves liver function, would help reduce the withdrawal time of lenvatinib administration in patients, thus transforming its local effect to prolonged TTP. The chemotherapeutic agent used in our study was idarubicin (10 mg) according to the dosage recommended by IDASPHERE phase II trial,^23^ suggested to be of lower hepatotoxicity than doxorubicin (150 mg) or epirubicin (50 mg), commonly used in previous studies. In addition, compared with conventional lipidol TACE, DEB‐TACE demonstrates a higher ORR, DCR, and lower all‐cause mortality with severe adverse events.^34^ Less hepatoxicity with the increase of tumor burden leaves the possibility for the patient to combine treatment with the standard first‐line systematic treatment or receive second‐line systematic treatment after disease progression, resulting in extended OS.^35^

Secreted by approximately 70% of patients with HCC, AFP is a well‐recognized tumor marker.^36^ High AFP level has been included as a negative factor in several existing prognostic scores for patients with advanced HCC.^37^, ^38^ PVTT is another common factor related to tumor burden, usually indicating a poor OS in patients with HCC.^3^ Our results indicate that, in the subgroup of patients with PVTT in second‐ or lower‐order portal vein branches, lenvatinib combined with IDADEB‐TACE conferred significant survival benefits compared with lenvatinib monotherapy, which was similar to a previous study, suggesting that TACE induced extensive intrahepatic tumor necrosis in the patients with intrahepatic PVTT.^39^ However, lenvatinib plus IDADEB‐TACE failed to improve survival in patients with first‐order portal vein branches PVTT. It might be because that lenvatinib decreases the collateral circulation of liver parenchyma coming from hepatic arteries, further deteriorating ischemic liver injury in patients with the first‐order portal vein branches PVTT.

Regarding the safety of the treatment, it should be noted that most AEs observed in our study were mild and manageable. Although the incidences of nausea/vomiting, fever, abdominal pain, and increased ALT/AST were more frequent in the LIDA than in the LEN group, these AEs were considered as post‐TACE syndrome^40^ rather than lenvatinib‐related AEs, and the incidences of other AEs were similar to those observed earlier.^5^, ^14^ In addition, these AEs neither led to more dose reduction nor interruption of lenvatinib in the LIDA group. Thereby, the toxicity profile of lenvatinib plus IDADEB‐TACE seems comparable to that of lenvatinib alone. Thus, IDADEB‐TACE does not exhibit additional toxicity when combined with lenvatinib to treat patients with advanced HCC.

There are some limitations in our study. First, as for the patients who have contraindications for TACE, their local treatment options are limited, remaining systematic treatment or supportive care. Our study had the limitation that the patients included cannot present all the patients with advanced‐stage HCC. Although the baseline characteristics of two groups were well matched after PSM, biases inherent to retrospective analysis were inevitable. Therefore, we have conducted a prospective randomized trial (ChiCTR2000034758) for more data support. Furthermore, the follow‐up time was short, which may also have skewed our data. Another limitation is that more than 80% of patients in our study were HBV positive; thereby, the efficacy of lenvatinib plus IDADEB‐TACE needs to be confirmed in HCC patients with HCV or other etiology. Last, patients with main PVTT were excluded because the contraindication of liver decompensation after TACE, which would not represent entire population with BCLC stage HCC.

## 5 CONCLUSION

Lenvatinib plus IDADEB‐TACE might be a potential first‐line therapy for patients with advanced HCC, with better survival advantages, tumor response, and acceptable toxicity in comparison with lenvatinib monotherapy.

## AUTHOR CONTRIBUTIONS

Guarantors of integrity: Wenzhe Fan and Jiaping Li; study concept and design: Wenzhe Fan, Bowen Zhu, and Jiaping Li; data acquisition: Shufan Yue, Xinlin Zheng, Xinhua Zou, Fuliang Li, Liangliang Qiao, Yanqin Wu, Miao Xue, Hongyu Wang, Yiyang Tang; data analysis, and interpretation: all authors; drafting of the manuscript:: Wenzhe Fan and Bowen Zhu; manuscript revision: Bowen Zhu and Jiaping Li; literature research: Wenzhe Fan and Bowen Zhu; clinical studies: all authors; approval of final version of submitted manuscript: all authors.

## CONFLICT OF INTEREST

All authors have no competing interest to declare.

## ETHICS STATEMENT

The study was approved by the Ethics Committee of Sun Yat‐sen University First Affiliated Hospital (Ethical number: [2020]256).

## Supporting information


Appendix S1

## Data Availability

The datasets used in this study are available from the corresponding author upon reasonable request.
