# Supplementary material for: Idarubicin‐Loaded DEB‐TACE plus Lenvatinib versus Lenvatinib for patients with advanced hepatocellular carcinoma: A propensity score‐matching analysis
Source: Cancer Med. 2022 Jun 13;12(1):61–72. doi: 10.1002/cam4.4937 (PMC9844616; doi:10.1002/cam4.4937)
Supplement: Supplementary file 1 — Appendix S1 [file CAM4-12-61-s001.docx]

**Supplemental Table 1. Causes of dose reduction or interruption of lenvatinib in the LIDA group and LEN group**

|  | **Reduction** | | **Interruption** | |
| --- | --- | --- | --- | --- |
|  | **LIDA Group (n=18)** | **LEN Group (n=15)** | **LIDA Group (n=14)** | **LEN Group (n=13)** |
| Hand-foot skin reaction | 1 | 1 | 1 | 0 |
| Diarrhea | 2 | 2 | 1 | 1 |
| Hypertension | 7 | 7 | 6 | 6 |
| Nausea/Vomiting | 2 | 1 | 1 | 1 |
| ALT/AST increased | 2 | 1 | 3 | 1 |
| Hyperbilirubinemia | 3 | 3 | 0 | 1 |
| Proteinuria | 1 | 0 | 1 | 2 |
| Decreased appetite | 0 | 0 | 1 | 1 |

Abbreviations: AST, aspartate aminotransferase; ALT, alanine aminotransferase; LIDA group, patients treated with lenvatinib plus idarubicin-loaded DEB-TACE; LEN group, patients treated with lenvatinib alone.


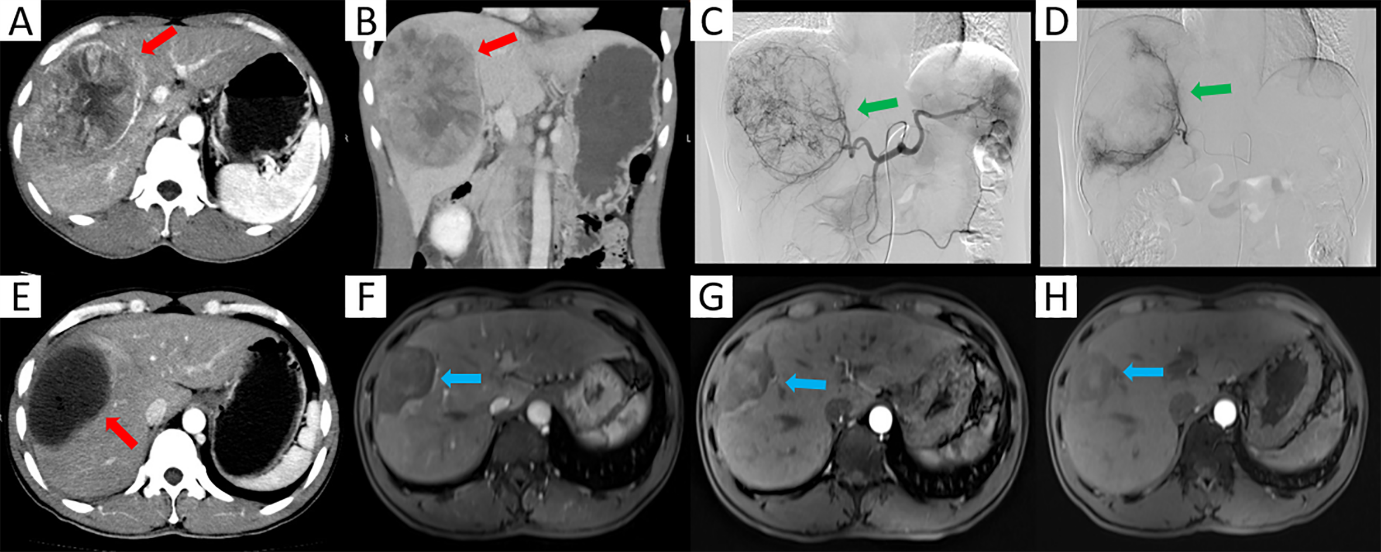


**Supplemental Figure 1: Computed tomography (CT), digital subtraction angiography (DSA), and magnetic resonance imaging (MRI) images of a 58-year-old man with hepatocellular carcinoma (HCC) who underwent one session of sorafenib combined with idarubicin-loaded drug-eluting beads transarterial chemoembolization (DEB-TACE).**

The red, green, and blue arrows indicate the main tumor on CT, DSA, and MRI, respectively. (A, B) Cross-sectional and coronal-sectional contrast-enhanced CT scans revealed a main tumor of 9.8 cm in the right lobe of the liver. (C, D) DSA images showing a rich arterial blood supply to the main tumor in the right lobe of the liver. (E–H) One cross-sectional contrast-enhanced CT and three T1 enhanced MRI scans performed at 1, 3, 6, and 12 months after the idarubicin-loaded DEB-TACE session, illustrating necrosis of the main tumor and significant shrinkage of the lesion, respectively.


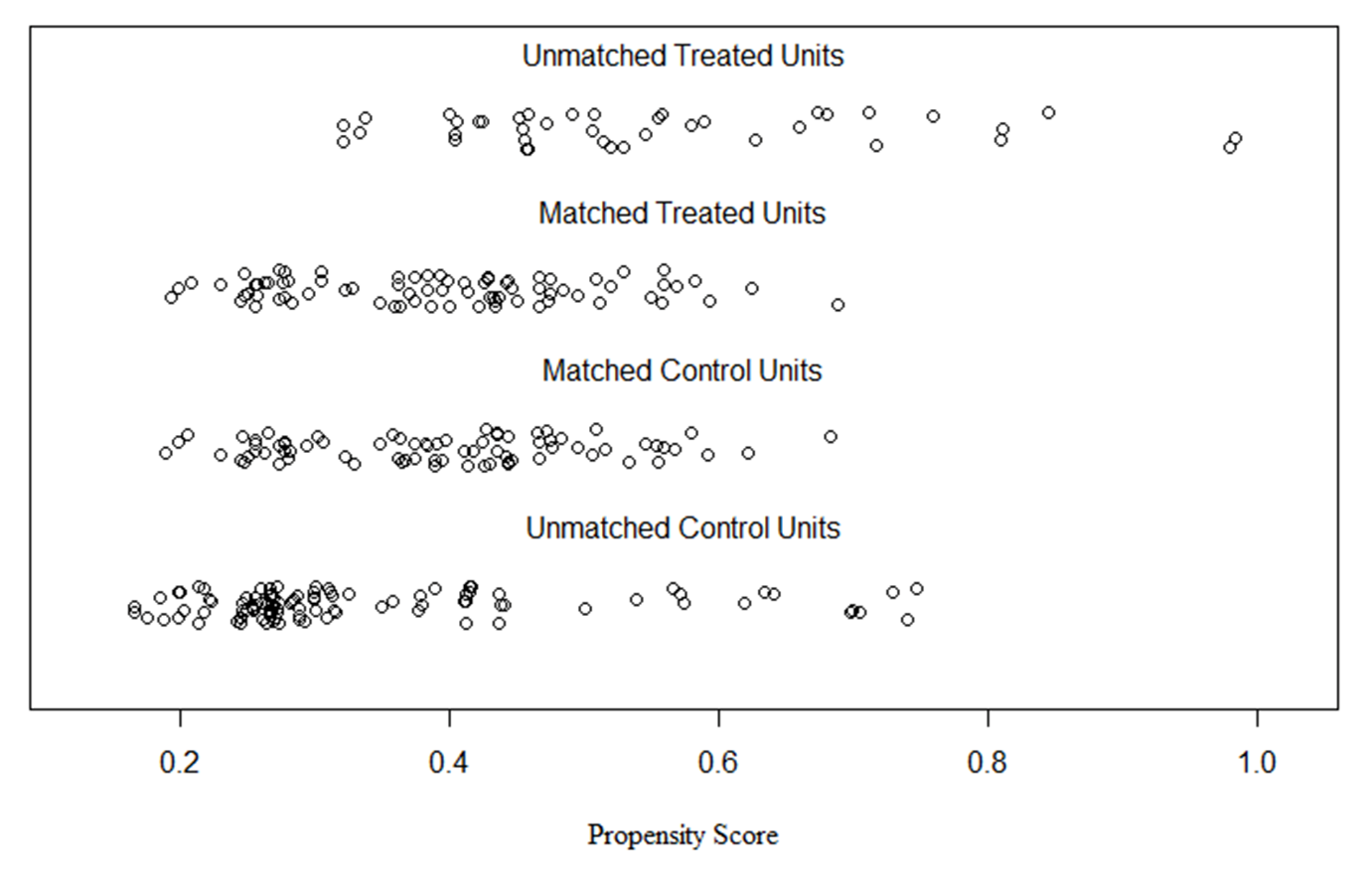


**Supplemental Figure 2: Jitter plot showing the propensity scores of the matched and unmatched samples in two group.**


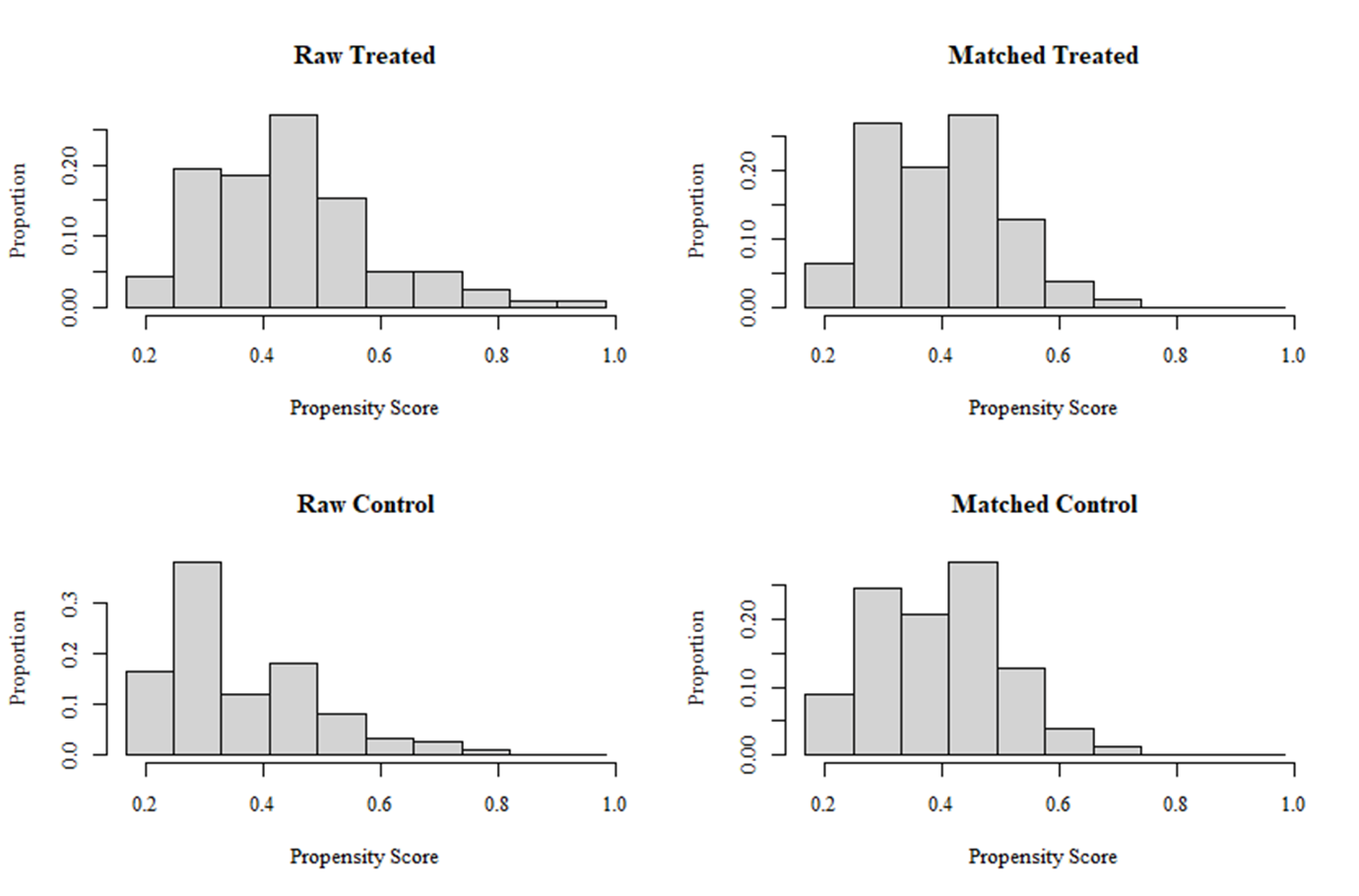


**Supplemental Figure 3: Histogram showing the initial and matched propensity scores in two group.**


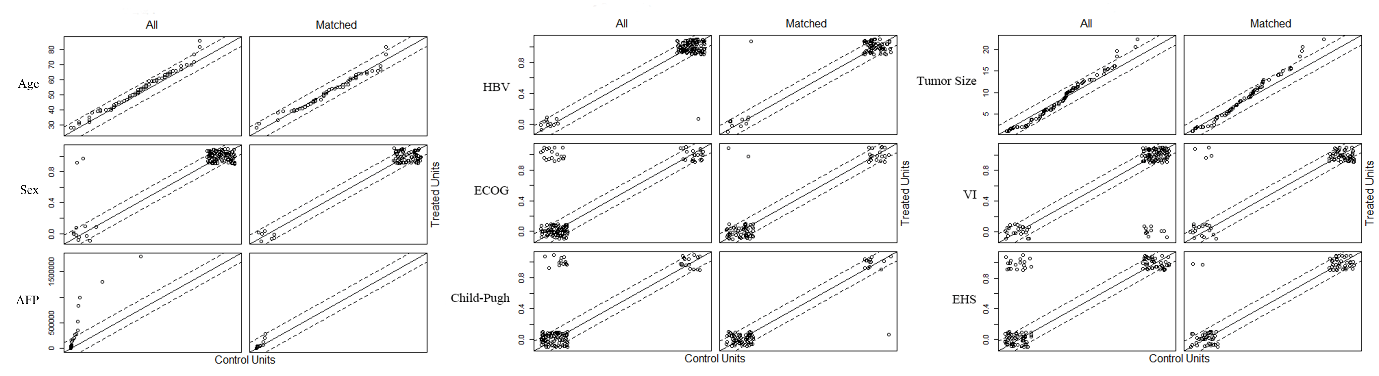


**Supplemental Figure 4: QQ plot showing the balance of each factor between two groups before and after propensity score-matching.**

AFP, alpha-fetoprotein; HBV, hepatitis B virus; ECOG, Eastern Cooperative Oncology Group (performance status); VI, vascular invasion; EHS, extrahepatic spread.


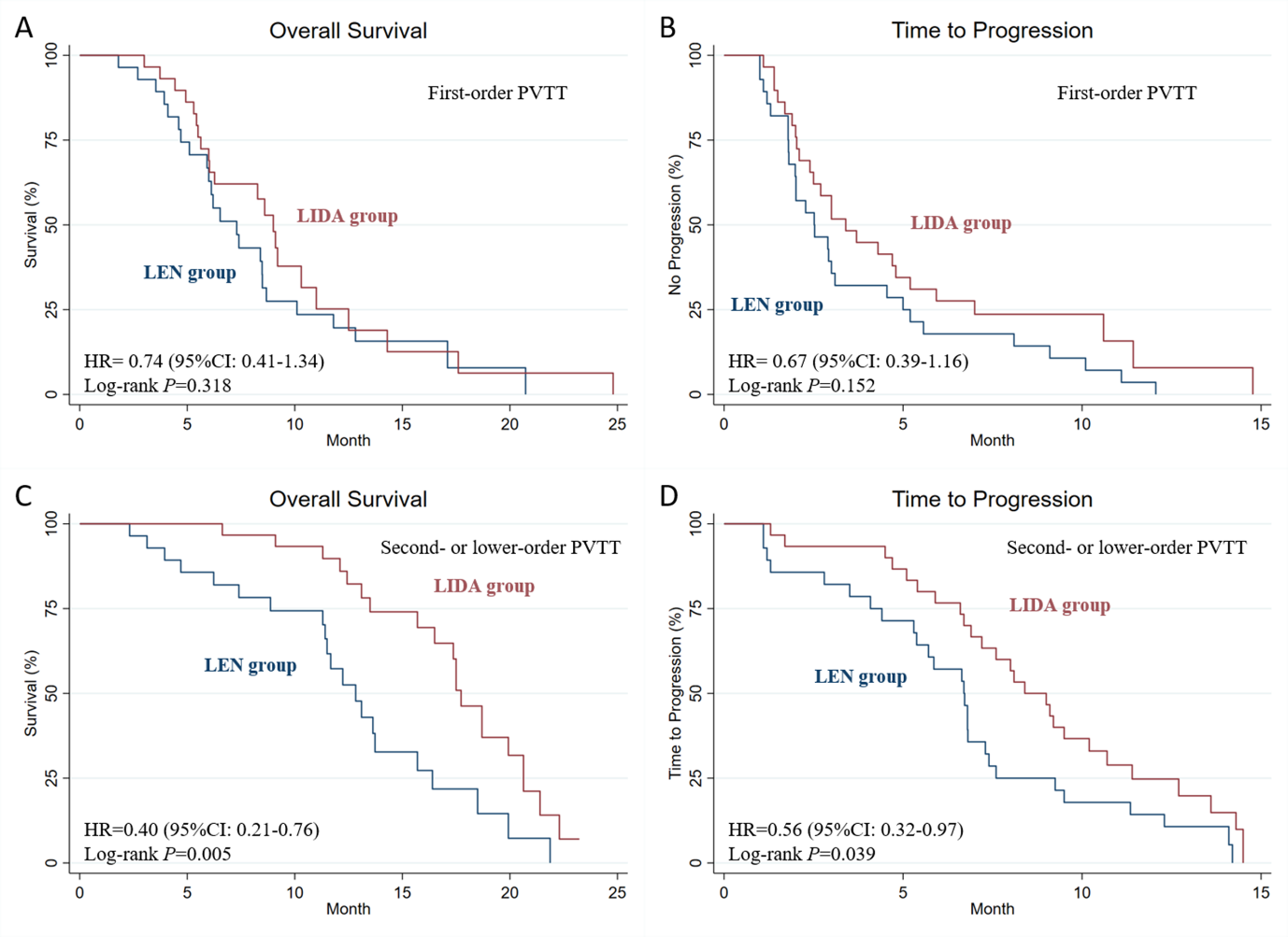


**Supplemental Figure 5: Kaplan-Meier curves showing overall survival and time to progression in patients with prognostic factor of with second- or lower-order and first-order PVTT in the LIDA group and in the LEN group.**

Dotted lines represent the 95% confidence interval. (A) Overall survival and (B) time to progression in patients with second- or lower-order PVTT. (C) Overall survival and (D) time to progression in patients with first-order PVTT.

HR, hazard ratio; CI, confident interval; PVTT, portal vein tumor thrombus; LIDA group, patients treated with lenvatinib plus idarubicin-loaded drug-eluting beads transarterial chemoembolization; LEN group, patients treated with lenvatinib alone.
